# Excessive Screen Time Among U.S. High School Students: Mental Health, Suicidal Ideation and Social Image Factors

**DOI:** 10.3390/healthcare13222833

**Published:** 2025-11-08

**Authors:** Satomi Imai, Austin Close, Tatiana Jones, Katherine Jones

**Affiliations:** 1 Center for Health Disparities, East Carolina University, Greenville, NC 27834, USA; 2 Department of Public Health, East Carolina University, Greenville, NC 27834, USAjonestati19@students.ecu.edu (T.J.)

**Keywords:** adolescent, screen time, digital media, television, mental health, suicidal ideation, protective factors

## Abstract

**Background/Objectives:** Adolescents’ screen time has increased alongside rising rates of depression and suicidal ideation and behavior. This study examined associations between excessive screen time and mental health among U.S. high school students, and explored whether social image factors (overweight perception, sports team participation, and academic performance) mitigate these associations. **Methods:** We analyzed data from the 2019 Youth Risk Behavior Surveillance System (YRBSS) (N = 13,677). Screen time was categorized as <2, 2–3, ≥4 h per day for television watching and digital device use (excluding school-related use). Multivariate logistic regression analyses estimated adjusted odds ratios (AORs) and 95% confidence intervals (CIs) for associations between screen time and mental health outcomes, adjusting for demographic and social image factors. **Results:** Overall, 31.7% of students reported using digital devices for ≥4 h per day, compared with 10.2% who watched television for ≥4 h per day. Using digital devices for ≥4 h per day was significantly associated with difficulty concentrating (AOR = 1.68), insufficient sleep (AOR = 1.43), feeling sad or hopeless (AOR = 1.86), and suicidal ideation (AOR = 1.69 for considering suicide; AOR = 1.77 for planning suicide) compared with using digital devices for <2 h per day, after adjusting for demographic and social image factors. Significant associations between television viewing and mental health outcomes appeared to be largely explained by social image factors. Female students and certain racial/ethnic groups were more likely to report mental health difficulties. Students with a positive social image reported lower odds of poor mental health outcomes and excessive screen time. **Conclusions:** Excessive digital device use is associated with poor mental health and suicidal ideation among high school students, with female students particularly vulnerable. Social image factors were also associated with screen time and mental health outcomes. Interventions that promote school engagement, sports team participation, and a positive social image may help mitigate the adverse effects of excessive screen time.

## 1. Introduction

Today’s adolescents face unprecedented challenges due to the rapid expansion of social media, online connectivity, and the emergence of generative artificial intelligence (AI). Screen time among high school students continues to rise as schoolwork, assignments, and textbooks require the use of tablets or computers with internet access. The use of digital devices has become deeply integrated into personal and social life [1]. Coinciding with the rise in digital engagement is an apparent increase in depressive symptoms and suicidal ideation among adolescents. Research has examined and demonstrated associations among high school students between elevated screen time and poor mental health [2,3,4,5] and suicidal ideation [6,7]. However, studies and reviews of recent studies show that these associations are often weak to modest in strength [8,9,10] or insignificant [11]. One challenge in interpreting these findings is that “screen time” encompasses a broad range of activities, including gaming, social media use, video streaming, chatting, and reading, among others. Each activity can have both positive and negative consequences, depending on factors such as the duration of daily use, the type of device, the nature of the content, and the mode of engagement (e.g., passive vs. active, solitary vs. social). Consequences can vary by specific age groups as well, due to developing brains and differences in social needs [12,13].

Many studies have reported difficulties in distinguishing the effects of different types of screen-based activities on adolescent mental health. For example, Boers et al. identified associations between hours spent on social media and depressive symptoms, but did not find the same association for video gaming [2]. This finding supported their hypothesis that excessive exposure to social media may evoke unrealistic social comparisons that may have negative effects on adolescents’ self-esteem and mental health, while video gaming activities may foster computer self-efficacy [2]. Furthermore, the impact of digital device use might not be uniform across individuals [14,15]. It interacts with multiple contextual factors, including school activities, peer relationships, and family circumstances. Some adolescents may experience significant negative effects, including addiction to certain social media sites and cyberbullying [16,17]. Others, however, may remain largely unaffected, or even experience positive effects, such as building broader social networks and developing digital skills. Reviewing studies on the relationship between social media use and depression or anxiety among both adults and adolescents, Lopes et al. noted that users’ manner of engagement (passive vs. active) and their coping mechanisms played a significant role in their experiences of depression or anxiety [18]. Using a large representative sample, Twenge and Farley found that social media use was more strongly associated with depressive symptoms, low self-esteem, and low life satisfaction than other online activities, particularly among girls [19]. Thus, in addition to excessive time spent on digital devices, the roles of digital literacy and protective strategies against negative effects must also be considered.

Despite these complexities, studies suggest that the total number of hours spent on screens per day is an important determinant of mental health outcomes [7,20]. Furthermore, reducing screen time, partly by replacing it with physical activities, may alleviate depressive symptoms [21,22,23], reinforcing the importance of achieving balance in daily activities. Excessive screen time may displace time for physical activity, in-person social interactions, and sleep, all of which are critical for adolescent well-being [24].

In this study, we focused on two primary questions: (1) whether hours spent watching television and using digital devices have differential effects on mental health and suicidal ideation and behavior, and, if so, what threshold of hours could be considered excessive and what are contributing variables for excessive screen time; and, (2) whether social image variables have interactive or independent effects that moderate the relationship between screen time and mental health outcomes and suicidal ideation and behavior. Few studies have directly compared hours spent watching television with hours spent using digital devices to distinguish the effects of digital media use from other sedentary behaviors. Further, no previous research has examined potential moderating factors related to screen time, poor mental health, and suicidal ideation using nationally representative data.

This study examined the effects of two components of screen-based behavior on mental health and suicidal ideation: (a) time spent using digital media and (b) time spent watching television. Television viewing is often considered a more passive activity compared to gaming or interactive online activities. We were interested in identifying how many hours should be considered excessive to affect mental health. While both television and digital media use typically involve sedentary behavior and limited in-person interaction, television may be more passive than social media use or gaming. Prolonged engagement in either form of screen time, however, may contribute to negative outcomes, including social isolation, disrupted sleep, and lower physical activity levels. These relationships may be bidirectional, as adolescents experiencing poor mental health may also turn to screens for distraction or escape.

We also examined protective or moderating factors that may buffer the potential negative effects of excessive screen time on mental health and suicidal ideation and behavior. Specifically, we explored indicators of adolescent social image, including perceived overweight status, academic performance, and participation in sports teams, as possible moderators. These factors were selected because they reflect critical aspects of adolescents’ social identity and school life, which can influence self-esteem and overall well-being. Research on screen time tends to isolate screen time hours from students’ school life and surrounding environments. This study examined screen time as part of students’ school life. Some research suggests that physical activity [25,26], participation in extracurricular activities [27] or having “green time” [28] may offer protective benefits, while poor body image associated with social media use has been linked to depressive symptoms [29,30].

This study used nationally representative data from the 2019 Youth Risk Behavior Surveillance System (YRBSS), which predates the COVID-19 pandemic. We used the 2019 YRBSS (rather than the 2021 or 2023 versions) because it was the most recent survey that included questions on screen time that distinguish between digital device use and television watching. The 2021 YRBSS asked a question on screen time, but the responses combined television watching with digital device use, including cellphones and computers. The 2023 YRBSS question on screen time asked only about social media use. Thus, the 2019 YRBSS was the latest available comprehensive U.S. youth dataset which included differentiated questions on both media types. Although screen time behaviors, particularly digital device use, and their potential negative impacts have likely increased since the pandemic [31,32] along with other changes in digital environments such as wider access to generative AI, these 2019 data serve as an important baseline for understanding pre-pandemic patterns and the implications for future trends.

By analyzing both risk and protective factors, our aim was to determine whether excessive screen time alone accounts for poor mental health outcomes or whether moderating factors play a significant role in shaping these associations. This study focused on high school students, whose brain development, social lives, academic demands, and sense of independence would differ from those of middle school students. This focus may allow us to provide more specific recommendations for the age group. We also included grade level as a variable, as 9th graders and 12th graders may differ significantly in their social development, cognitive maturity and school roles.

Mental health problems can manifest internally, such as through anxiety and depression, and externally through behavioral problems such as drug use or smoking. This study focused on internalizing forms, including mental health problems (difficulty in concentration, insufficient sleep, and feeling sad or hopeless and) and suicidal ideation and behavior.

We hypothesized that longer screen time would be associated with poorer mental health and increased suicidal ideation and behavior. We predicted that this association would be stronger among 9th–10th graders, whose identity formation and independence are still emerging, and among female students, as previous studies have indicated. We also expected that positive social image variables would moderate the negative effects of prolonged screen time. We further predicted that the associations with negative outcomes would be weaker for television viewing than for digital device use, given the differing levels of engagement and content exposure between the two forms of media. Finally, we predicted distinct patterns of characteristics for excessive screen time by demographic and social image variables so that female students, those who had poor social images, would be more likely to report excessive screen time. See Supplementary Material for the STROBE checklist used in this study.

## 2. Materials and Methods

### 2.1. Data Source and Population

We analyzed data from U.S. high school students in grades 9–12 who participated in the 2019 National YRBSS survey (N = 13,677). The YRBSS is conducted by the Centers for Disease Control and Prevention (CDC) every two years among U.S. middle and high school students using a school-based, cross-sectional design, including three-stage cluster-sampling selected schools, classes, and students, with weights applied to adjust for nonresponse and ensure representativeness by sex, grade, and race/ethnicity. The data are nationally representative, adjusted for population characteristics (accounting for nonresponse and oversampling of Black and Hispanic students), and publicly available. The survey focuses on physical and mental health and behavioral risk factors. Participation is voluntary and anonymous. It is classified as a public health surveillance activity, exempt from full IRB review, and schools obtain parental permission following either passive or active parental permission procedures, depending on district or state policy [33]. The completed STROBE checklist is provided in Supplementary Material (Table S1).

### 2.2. Outcome Measures

The dependent variables were mental health indicators and suicidal ideation and behavior. Mental health variables included: (1) Had difficulty concentrating: Responses of “Yes” to the question, “Because of a physical, mental, or emotional problem, do you have serious difficulty concentrating, remembering, or making decisions?”; (2) Slept 5 h or less: Responses of “4 or less” or “5 h” to the question, “On an average school night, how many hours of sleep do you get?”; (3) Felt sad or hopeless: Responses of “Yes” to the question, “During the past 12 months, did you ever feel so sad or hopeless almost every day for two weeks or more in a row that you stopped doing some usual activities?”

Suicidal ideation and behavior included: (1) Considered suicide: Responses of “Yes” to the question, “During the past 12 months, did you ever seriously consider attempting suicide?”; (2) Planned suicide: Responses of “Yes” to the question, “During the past 12 months, did you make a plan about how you would attempt suicide?”; (3) Attempted suicide: Responses of “one or more times” to the question, “During the past 12 months, how many times did you actually attempt suicide?”

### 2.3. Independent Variables

The independent variables included: (1) Television watching: Categorized as less than 2, 2–3, or 4 or more hours per day, based on responses to “On an average school day, how many hours do you watch TV?” The response categories were: “1. I do not watch TV on an average school day”; “2. Less than 1 h per day”; “3. 1 h per day”; “4. 2 h per day”; “5. 3 h per day”; “6. 4 h per day”; “7. 5 or more hours per day.”; (2) Daily screen time on digital devices (excluding schoolwork): Categorized as less than 2, 2–3, or 4 or more hours per day, based on responses to “On an average school day, how many hours do you play video or computer games or use a computer for something that is not school work? (Count time spent playing games, watching videos, texting, or using social media on your smartphone, computer, xbox, playstation, ipad, or other tablet).” The response categories were: “1. I do not play video or computer games or use a computer for something that is not school work”; “2. less than 1 h per day”; “3. 1 h per day”; “4. 2 h per day”; “5. 3 h per day; 6. 4 h per day”; “7. 5 or more hours per day.”

### 2.4. Demographic and Moderating Variables

The demographic variables included: (1) Sex (female, male); (2) Race/ethnicity (non-Hispanic White, non-Hispanic Black, Hispanic, non-Hispanic Other); (3) Grade (9th, 10th, 11th, 12th).

The moderating factors (social image variables) included: (1) Overweight perception: Responses of “slightly overweight” or “very overweight” to the question, “How do you describe your weight?”; (2) Participation in team sports: Responses of “1”, “2”, “3 or more teams” based on “During the past 12 months, on how many sports teams did you play?”; (3) Academic grades: Responses of “mostly As or Bs” to the question, “During the past 12 months, how would you describe your grades in school?”

### 2.5. Statistical Analyses

All analyses were conducted using SAS version 9.4 (SAS Institute Inc., Cary, NC, USA). Multivariate logistic regression analyses were performed for three mental health variables and three suicidal ideation and behavior variables by hours of television watching and digital device use, including three social image variables. An additional multivariate logistic regression analysis was performed to identify risk factors associated with spending 4 or more hours daily on screen time.

Logistic regression was used to estimate adjusted odds ratios (AORs) and 95% confidence intervals (CIs) for all binary outcomes. Weighted analyses were conducted using the PROC SURVEYFREQ and PROC SURVEYLOGISTIC procedures in SAS. Odds ratios (ORs) were adjusted for demographic variables (sex, grade, and race/ethnicity) in Model 1, and additionally for screen time and social image variables (overweight perception, sports team participation, and academic grades) in Model 2. Interaction terms between screen time and social image variables were included in Model 2, and corresponding *p*-values are reported in the Results. Percentages and 95% confidence intervals (CIs) represent weighted estimates obtained using SAS survey procedures. Analyses involved survey-weighted estimates with standard errors (SEs) adjusted for strata and primary sampling unit (PSU) design using Taylor series linearization. For multivariate logistic regression analyses, cases with missing data were excluded using listwise deletion.

Although odds ratios may be overestimated for common outcomes with prevalence greater than 30%, logistic regression was chosen rather than Poisson regression for risk ratios to maintain consistency in the results, as many of our outcome measures had a prevalence of ≤20%. AORs were interpreted as measures of association while acknowledging the potential overestimation of risk for common outcomes.

We applied multiple-testing adjustments using the Benjamini–Hochberg false discovery rate (FDR) procedure (PROC MULTTEST) for the main effects of each hypothesis. We included both raw *p*-values and adjusted *p*-values in tables and used adjusted *p*-values for interpretation. No FDR adjustments were applied to interaction terms; only unadjusted *p*-values are reported in the Results and any significant interactions were considered exploratory. The alpha level was set at 0.05. 

## 3. Results

### 3.1. Participant Characteristics

A total of 13,677 high school students participated in the 2019 YRBSS. Among them, 49.37% (n = 6690) were female and 50.63% (n = 6862) were male (missing data: n = 125). Regarding race/ethnicity, 51.18% were non-Hispanic White (n = 6784), 12.20% were non-Hispanic Black (n = 2040), 26.11% were Hispanic (n = 3038, including mixed Hispanic races), and 10.51% were non-Hispanic Other (n = 1393, including non-Hispanic mixed races) (missing data: n = 422).

### 3.2. Screen Time

Overall, 20.64% (95% CI: 19.70–21.59) of students reported spending 5 or more hours per day using digital devices, while 5.84% (95% CI: 5.29–6.38) reported watching television for the same duration. A higher proportion of students reported using digital devices for 4 or more hours per day (31.71%, 95% CI: 29.64–31.78) compared with those watching television for 4 or more hours per day (10.17%, 95% CI: 9.48–10.88). Preliminary multivariate logistic regression analyses suggested significant associations for both 4 or more and 5 or more hours of daily screen time. However, a 5-h cut-off yielded sample sizes for television watching that were too small for reliable subgroup analysis; thus, a 4-h cut-off was selected and used throughout, with sensitivity analyses confirming the robustness of this choice.

### 3.3. Screen Time and Mental Health

Had Difficulty Concentrating. As shown in Table 1, Model 1 multivariate logistic regression analyses, adjusted for demographic variables (sex, grade level, and race/ethnicity), produced adjusted odds ratios (AORs) for mental health variables by television watching and digital device use. AORs for sex, race/ethnicity, and grade level indicated significant associations. Adjusted *p*-values were calculated to control for multiple comparisons for each hypothesis and were used in interpreting the results. Female students, compared to male students, had higher odds of reporting difficulty concentrating (AOR = 2.00, 95% CI: 1.86–2.17). Non-Hispanic Black students (AOR = 1.71, 95% CI: 1.42–2.07) and students of other races (AOR = 1.39, 95% CI: 1.16–1.65) had higher odds compared to non-Hispanic White students. Similarly, 11th graders (AOR = 1.46, 95% CI: 1.21–1.75) and 12th graders (AOR = 1.56, 95% CI: 1.30–1.88) had higher odds compared to 9th graders. Both television watching and digital device use for 4 or more hours were significantly associated with difficulty concentrating (AOR = 1.38, 95% CI: 1.17–1.63; AOR = 1.91, 95% CI: 1.67–2.18, respectively).

Model 2, after adjusting for all the variables, including demographic, screen time, and social image variables, showed that females were 2.17 times more likely than males to report difficulty concentrating (AOR = 2.17, 95% CI: 1.97–2.40). Compared to non-Hispanic White students, non-Hispanic Black students had lower odds (AOR = 0.67, 95% CI: 0.54–0.83). Students of other races reported higher odds (AOR = 1.22, 95% CI: 1.01–1.47), but this was only marginally significant after adjustment for multiple tests (adjusted *p* = 0.0627). In Model 2, the previously significant AORs for grade level became non-significant. Television watching did not show significant differences overall. Students who used digital devices for 4 or more hours reported higher odds of difficulty concentrating than those who used them for less than 2 h (AOR = 1.68, 95% CI: 1.47–1.92). All social image variables were significant: perceived overweight (AOR = 1.30, 95% CI: 1.14–1.49), sports team participation (AOR = 1.35, 95% CI: 1.22–1.49), and academic grades (AOR = 2.19, 95% CI: 1.87–2.56).

None of the interactions with social image variables were significant except for the interaction between perceived overweight and television watching. The significant interactions suggested that students who watched TV for 2–3 h or more than 4 h were more likely to report difficulty concentrating (raw *p* = 0.0346 and raw *p* = 0.017, respectively).

Sleep Hours. Model 1 showed significant AORs for sex, race/ethnicity, and grade level. Female students, compared to male students, (AOR = 1.14, 95% CI: 1.01–1.28), non-Hispanic Black students (AOR = 1.48, 95% CI: 1.42–2.07) and students of other races (AOR = 1.39, 95% CI: 1.16–1.65) compared to non-Hispanic White students, and 11th (AOR = 1.46, 95% CI: 1.21–1.75) and 12th graders (AOR = 1.56, 95% CI: 1.30–1.88) compared to 9th graders had significant AORs. There were significant associations with television watching for 2–3 h (AOR = 0.79, 95% CI: 0.69–0.90) and digital device use (AOR = 1.45, 95% CI: 1.20–1.76). Compared to those who watched television for less than 2 h, those who watched for 2–3 h were less likely to sleep 5 h or less.

In Model 2, female students (AOR = 1.22, 95% CI: 1.07–1.38), non-Hispanic Black students (AOR = 1.48, 95% CI: 1.23–1.77), and students of other races (AOR = 1.37, 95% CI: 1.15–1.64), as well as 11th (AOR = 1.56, 95% CI: 1.27–1.92) and 12th graders (AOR = 1.66, 95% CI: 1.38–1.98), also showed significant AORs as in Model 1. Students who watched television for 2–3 h had a lower AOR (AOR = 0.78, 95% CI: 0.68–0.88) for sleeping 5 h or less compared to those who watched television for less than 2 h, while no difference was found for those watching 4 or more hours. In contrast, students who used digital devices for 4 or more hours had higher adjusted odds of sleeping 5 h or less (AOR = 1.43, 95% CI: 1.21–1.68). All social image variables were associated with short sleep duration: overweight perception (AOR = 1.36, 95% CI: 1.20–1.53), not participating in sports teams (AOR = 1.42, 95% CI: 1.23–1.64), and lower academic grades (AOR = 2.19, 95% CI: 1.89–2.53). The only significant interaction was between sports team participation and television watching hours (significant among students who did not participate in sports teams and watched television for 4 or more hours, raw *p* = 0.0024).

Felt Sad or Hopeless. Model 1 showed significant odds ratios for sex, race/ethnicity, grade level, and screen time. Female students (AOR = 2.40, 95% CI: 2.19–2.64), non-Hispanic Black students (AOR = 0.82, 95% CI: 0.71–0.96), Hispanic students (AOR = 1.19, 95% CI: 1.06–1.34), 11th graders (AOR = 1.22, 95% CI: 1.05–1.40), and 12th graders (AOR = 1.28, 95% CI: 1.07–1.53) showed significant associations. Both television watching and digital device use for 4 or more hours were also significantly associated (AOR = 1.33, 95% CI: 1.10–1.61; and AOR = 2.00, 95% CI: 1.72–2.33, respectively). Digital device use for 2–3 h was marginally significant after multiple testing adjustment (adjusted *p* = 0.0551).

In Model 2, after adjusting for all variables, female students (AOR = 2.43, 95% CI: 2.16–2.74), non-Hispanic Black students (AOR = 0.78, 95% CI: 0.64–0.95), and 11th graders (AOR = 1.22, 95% CI: 1.05–1.42) showed significant associations. Television watching was not associated with feeling sad or hopeless, whereas digital device use was significantly associated at both 2–3 h (AOR = 1.24, 95% CI: 1.05–1.47) and 4 or more hours (AOR = 1.86, 95% CI: 1.55–2.23). All social image variables were significant: overweight perception (AOR = 1.32, 95% CI: 1.17–1.49), sports team participation (AOR = 1.34, 95% CI: 1.19–1.50), and academic grades (AOR = 1.74, 95% CI: 1.53–1.98). None of the interaction terms were significant.

### 3.4. Screen Time and Suicidal Ideation and Behavior

As shown in Table 2, Model 1 showed significant associations between sex, race/ethnicity, and suicidal ideation. Significant associations were observed for considering suicide (AOR = 2.09, 95% CI: 1.85–2.36 for females), planning suicide (AOR = 1.94, 95% CI: 1.74–2.16 for females), and attempting suicide (AOR = 1.76, 95% CI: 1.39–2.22 for females; and AOR = 1.57, 95% CI: 1.16–2.12 for non-Hispanic Black students).

There were also significant associations between television watching for 4 or more hours and considering suicide (AOR = 1.33, 95% CI: 1.10–1.61) and attempting suicide (AOR = 1.46, 95% CI: 1.12–1.90). For digital device use of 4 or more hours, all AORs were significant for considering (AOR = 2.00, 95% CI: 1.72–2.33), planning (AOR = 1.87, 95% CI: 1.57–2.23), and attempting suicide (AOR = 1.54, 95% CI: 1.21–1.97).

In Model 2, female students had significantly higher AORs for considering (AOR = 2.17, 95% CI: 1.85–2.54), planning (AOR = 2.05, 95% CI: 1.78–2.36), and attempting suicide (AOR = 1.90, 95% CI: 1.43–2.51) compared to male students, after adjusting for all variables. For considering suicide, Hispanic students (AOR = 0.71, 95% CI: 0.58–0.88) had a significantly lower AOR compared to non-Hispanic White students. Racial differences were also significant for planning suicide: Hispanic students showed a lower AOR (AOR = 0.75, 95% CI: 0.61–0.92), and students of other races had a higher AOR (AOR = 1.31, 95% CI: 1.06–1.63) compared to non-Hispanic White students. For attempting suicide, students of other races (AOR = 1.53, 95% CI: 1.10–2.13) showed a significantly higher AOR, while non-Hispanic Black students had a marginally higher AOR after adjusting multiple testing (AOR = 1.44, 95% CI: 1.06–1.98, adjusted *p* = 0.0506). None of the grade-level associations were significant for either Model 1 or Model 2 across all suicidal ideation and behavior variables.

For screen time variables, in Model 1 students who reported watching television for 4 or more hours had a higher AOR for considering suicide (AOR = 1.33, 95% CI: 1.10–1.61) compared to those with less than 2 h. Those using digital devices for 4 or more hours had twice the odds of having considered suicide (AOR = 2.00, 95% CI: 1.72–2.33) compared to those with less than 2 h of use. For planning suicide both in Model 1 and Model 2 those who used 4 h or more of digital device had higher AORs compared with those who used them for less than 2 h (AOR = 1.87, 95% CI: 1.57–2.23, for Model 1; and AOR = 1.77, 95% CI: 1.44–2.18 for Model 2). For attempting suicide, both television watching and digital device use for 4 h or more in Model 1 were significantly associated (AOR = 1.46, 95% CI: 1.12–1.90 for television; and AOR = 1.54, 95% CI: 1.21–1.97 for digital devices). However, none of associations remained significant in Model 2.

All social image variables were significant for considering suicide: overweight perception (AOR = 1.67, 95% CI: 1.43–1.95), not participating in sports teams (AOR = 1.37, 95% CI: 1.15–1.63), and lower academic grades (AOR = 1.68, 95% CI: 1.49–1.89). For planning and attempting suicide, all social image variables except sports team participation were significant. For planning suicide, overweight perception (AOR = 1.61, 95% CI: 1.38–1.88) and lower academic grades (AOR = 1.81, 95% CI: 1.52–2.14) were associated with increased odds. For attempting suicide, overweight perception (AOR = 1.60, 95% CI: 1.31–1.95) and lower academic grades (AOR = 2.53, 95% CI: 1.99–3.23) were also associated with increased odds. Sports team participation was marginally significant for planning and attempting suicide after multiple-testing adjustment (adjusted *p* = 0.076 and 0.0559, respectively).

### 3.5. Screen Time and Social Image Variables

Table 3 presents the multivariate logistic regression results for excessive screen time (4 or more hours), examining both television watching and digital device use. Risk factors were assessed by demographic and social image variables. AORs were adjusted for all the variables, including demographic and social image variables. For television watching, female students were more likely to watch television for 4 or more hours (AOR = 1.26, 95% CI: 1.07–1.48). Students who were non-Hispanic Black (AOR = 3.01, 95% CI: 2.45–3.71), Hispanic (AOR = 1.30, 95% CI: 1.18–1.77), and of other races (AOR = 1.54, 95% CI: 1.07–2.23) had higher odds of watching television for 4 or more hours compared with non-Hispanic White students. Eleven graders were less likely to watch television compared to 9th graders (AOR = 0.77, 95% CI: 0.61–0.97).

All social image variables showed significant associations. Students who perceived themselves as overweight were more likely to watch television for 4 or more hours (AOR = 1.14, 95% CI: 1.02–1.27) than those who did not. Students who did not participate in sports teams had higher odds of watching television for 4 or more hours (AOR = 1.37, 95% CI: 1.15–1.63), as did students with lower academic grades (AOR = 1.78, 95% CI: 1.41–2.23) compared to those who participated in team sports or had higher grades, respectively.

For digital device use, no significant associations were found for sex, race/ethnicity, or grade level after adjusting for all variables. All social image variables were significantly associated with prolonged digital device use: overweight perception (AOR = 1.44, 95% CI: 1.29–1.60), not participating in sports teams (AOR = 1.68, 95% CI: 1.44–1.97), and lower academic grades (AOR = 1.48, 95% CI: 1.30–1.68).

## 4. Discussion

**Figure 1:**
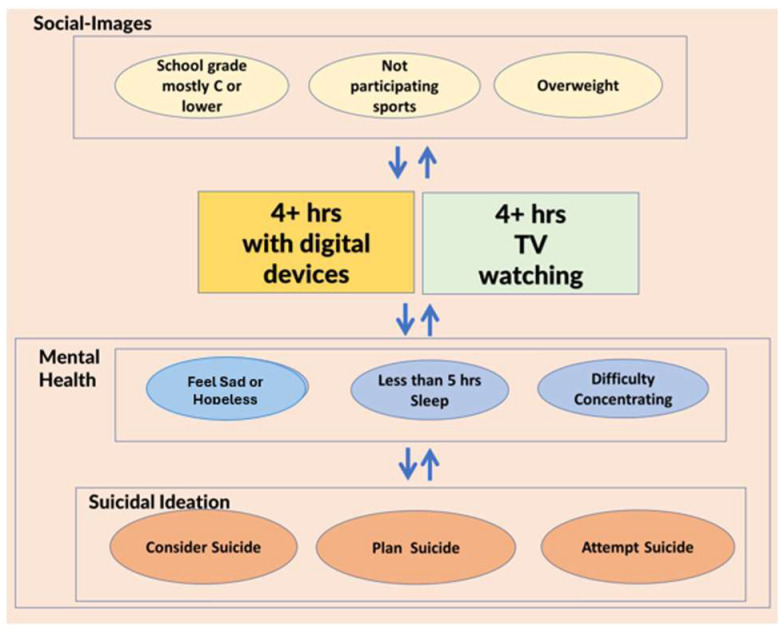
Conceptual model of the relationships among screen time, mental health, suicidal ideation and behavior, and social image factors.

As shown in Figure 1, this study examined associations between excessive screen time on television and digital devices, mental health, suicidal ideation and behavior, and social images among U.S. high school students, using nationally representative data from the 2019 YRBSS. Our objectives were to: (1) examine differences in associations with mental health and suicidal ideation and behavior between excessive hours spent watching television and using digital devices; and (2) further, examine moderating effects of social image factors. In Multivariate Model 1, we examined the associations between screen time and mental health and suicidal ideation and behavior while adjusting for demographic variables. In Multivariate Model 2, we further adjusted for social image factors to examine their associations in addition to those of screen time variables. 

In Multivariate Model 1, both prolonged television watching and digital device use were associated with negative mental health outcomes, except for television viewing and insufficient sleep. Students who reported watching television for 2–3 h were less likely to have insufficient sleep (AOR = 0.79) compared to those who watched less than 2 h. This effect remained significant in Multivariate Model 2 after social image variables were accounted for. This finding suggests that a moderate amount of daily television viewing may provide relaxation or family interaction time. Alternatively, students who watched less than 2 h may have engaged in other activities that reduced their sleep. All other effects were associated with prolonged screen time and adverse mental health outcomes, as well as suicidal ideation and behavior.

Watching 4 or more hours of television per day was linked to difficulty concentrating, feeling sad or hopeless, and suicidal ideation and suicide attempts, but not to suicidal planning. Spending 4 or more hours on digital devices consistently had adverse effects on all mental health outcomes, and suicidal ideation and behavior in Model 1. However, when social image variables were included in Model 2, several significant effects disappeared, suggesting that the effects of prolonged screen time were partly explained by social image variables. Specifically, for television watching, most significant associations disappeared except for the positive association with sleep. In contrast, for prolonged digital device use, associations with all mental health measures remained significant, suggesting independent effects beyond social image variables. For suicidal ideation and behavior, all associations remained significant except for suicide attempts. Thus, these differences in Model 1 and 2 for associations between television watching and digital device use suggest the effects from their prolonged engagements differ; negative effects of prolonged television watching are mostly explained by social image variables, whereas those of prolonged digital device use might independently influence mental health and suicidal ideation apart from social image variables (Table 1 and Table 2).

Importantly, our study demonstrates that social image factors, including perceived overweight status, sports team participation, and academic performance, moderated the relationship between screen time and mental health outcomes and suicidal ideation and behavior. Adolescents who perceive themselves as overweight report higher rates of sadness or hopelessness and suicidal ideation and behavior. Conversely, high academic performance and participation in sports teams appear protective, mitigating the negative effects of excessive screen time. These findings suggest that interventions promoting positive social images, physical activity, and school engagement could buffer the negative effects of prolonged screen time while also helping to reduce screen time. At the same time, promoting digital literacy is essential [34,35].

The stronger and more consistent negative associations observed for digital device use, compared with television viewing, may reflect inherent differences between these media, not just the amount of daily usage. Digital media’s interactive features can increase exposure to social comparison, cyberbullying, and other stressors, whereas television, being more passive, may affect health mainly through displacement of physical activity and, in moderation, may provide relaxation.

Multivariate analyses examining associations between 4 or more hours of screen time and demographic and social image variables also revealed differences between the two media (Table 3). Both prolonged television watching and digital device use were significantly associated with social image variables, suggesting that a positive social image may protect against excessive screen time. The demographic profiles of users differed. More females, non-Hispanic Black, Hispanic, and students of other racial/ethnic groups, as well as students in lower grades, watched television for 4 or more hours compared with males, non-Hispanic White students, and 12th graders. In contrast, there were no significant demographic associations for 4 or more hours of digital device use when social image variables were adjusted. The two media differ in their relation to mental health and suicidal ideation, both in terms of context of use and types of content, and one medium may have been particularly attractive to certain students. We calculated the correlation between the number of hours spent on the two media, which was significant but low (*r* = 0.11, *p* < 0.0001), suggesting limited overlap between the groups of students with prolonged screen time.

For all the outcome measures in both Model 1 and Model 2, female students showed significantly higher AORs than male students, suggesting a greater vulnerability to poor mental health and greater risk for suicidal ideation and behavior. The results by race/ethnicity showed mixed patterns. Non-Hispanic White students showed significantly higher AORs for considering and planning suicide. Non-Hispanic Black students had significantly higher AORs for having insufficient sleep but lower AORs for difficulty concentrating, feeling sad or hopeless, or considering suicide compared to non-Hispanic White, although they showed a significantly higher AOR for attempting suicide. Hispanic students were less likely to consider or plan suicide compared to non-Hispanic White students. Students of other races showed more vulnerability; they were more likely to have difficulty concentrating, insufficient sleep, and were more likely to consider, plan and attempt suicide compared to non-Hispanic White students.

There were no differences between race/ethnicities in digital device use for 4 or more hours. Students of other races showed difficulty concentrating while non-Hispanic Black students showed fewer issues with concentrating and feeling sad or hopeless but reported insufficient sleep in Model 2. These findings reinforce prior research suggesting that excessive screen time can negatively impact adolescent mental health [2,4,32] but highlight sex differences in vulnerability. Female students appear more susceptible to the negative effects of excessive digital device use, particularly regarding feelings of sadness or hopelessness and suicidal ideation. These sex differences may reflect psychosocial factors, including body image concerns, social pressures, or differences in online activity types (e.g., social media vs. gaming).

Associations between grade level and mental health measures were found for difficulty concentrating for Model 1, but these were not significant in Model 2, suggesting that screen time or social image variables accounted for the differences. However, insufficient sleep was observed for 11th and 12th graders in both Model 1 and Model 2, suggesting they had independent problems of insufficient sleep. For feeling sad or hopeless, in Model 1, 11th and 12th graders had significantly higher AORs, while in Model 2, the AOR remained significant for 11th graders when all the variables were controlled. None of associations between grade level and suicidal ideation and behavior were significant. Contrary to our hypothesis, it was not the younger students, but the 11th and 12th graders who showed mental health issues, insufficient sleep and feeling sad or hopeless. This might be attributable to their stage in independence and pressure from schoolwork and other activities.

Our study has several strengths, including the use of a large, nationally representative sample, evaluation of multiple mental health outcomes, and consideration of social image as a moderating factor. Overall, these findings underscore the importance of considering both the quantity and context of adolescent screen use. Interventions should focus not only on limiting screen time but also on enhancing protective factors such as physical activity, positive social image, and academic engagement. Healthcare providers, educators, and parents should be aware of the mental health risks associated with excessive digital device use, particularly for female adolescents and those with negative self-perceptions; they should consider multifaceted approaches that address both screen exposure and underlying psychosocial factors to reduce the risk of depression and suicidal ideation and behaviors in adolescents.

A post-pandemic study using the 2021–2023 National Health Interview Survey–Teen (NHIS–Teen) found that a striking 50.4% of youth aged 12–17 spent 4 or more hours per day on screen-based activities [1]. Although these data are not directly comparable, our results showed that 31.7% reported daily digital device use and 10.2% reported daily television viewing. We compared television watching and digital device use; however, time spent on traditional television is likely to continue declining as television and movie content become increasingly accessible via digital platforms, shifting viewing habits toward more individualized and private consumption. This study may remain a benchmark for pre-pandemic associations between screen time, mental health, and suicidal ideation among U.S. high school students.

Limitations and Future Research. This study has several limitations. First, the cross-sectional design of the YRBSS data precludes causal inference. While excessive screen time is associated with poor mental health and suicidal ideation and behavior, the directionality of these relationships cannot be determined. Adolescents experiencing depressive symptoms may also engage in higher screen time as a coping mechanism. Second, all measures, including screen time, television viewing, mental health indicators, and social image factors, were self-reported, which may introduce recall or social desirability bias. Also, our results relied on single-item, self-reported questions for mental health and suicidal ideation and behavior, which may introduce measurement error and are therefore less precise than multi-item scales. In addition, because odds ratios may overstate relative risk for outcomes with prevalence above 30%, the magnitude of associations should be interpreted with caution, though the direction and relative strength of effects remain informative [36,37]. Third, categorizing screen time by hours does not capture the type or quality of content consumed, nor the context of usage (e.g., solitary vs. social, or interactive vs. passive), which may differentially influence mental health. Fourth, data were collected prior to the COVID-19 pandemic. Post-pandemic changes in digital behavior, including increased reliance on online learning, and the introduction of new technologies such as AI-driven social media, may have intensified screen exposure and its associated risks. Therefore, our findings may not fully reflect post-pandemic adolescent experiences. Finally, while this study examined several protective social image factors, other important moderators, such as family environment, peer relationships, or socioeconomic status, were not included and might have influenced the associations between screen time and mental health outcomes.

Because digital device use encompasses a wide range of activities, large-scale, representative surveys are needed to examine screen time patterns with particular focus on content, interactivity, and context in relation to both mental and physical health outcomes. In addition, longitudinal, experimental, and program-evaluation studies are required to capture the ongoing effects of evolving digital environments [38,39,40]. Given the rapid advancement of AI technologies, qualitative research will also be critical to identify emerging challenges faced by youth. Future studies should further explore interventions that strengthen protective factors, such as physical activity, academic engagement, positive social image, and digital literacy, and evaluate their effectiveness in mitigating screen time-related mental health risks. Such efforts are essential to inform evidence-based strategies for adolescent mental health and suicide prevention in the digital era.

## 5. Conclusions

Excessive digital device use is significantly associated with poor mental health and suicidal ideation among U.S. high school students, with female students particularly vulnerable. Social image factors, including perceived overweight status, academic performance, and sports team participation, were independently associated with these outcomes and may mitigate the adverse effects of excessive screen time. The negative associations between prolonged television watching and mental health measures and suicidal ideation were no longer significant after adjusting for social image variables, suggesting those relationships were partially explained by social image variables. These findings suggest that interventions aimed at reducing excessive screen time, promoting a positive social image, encouraging academic engagement, and increasing participation in team sports could be effective strategies to support adolescent mental health and suicide prevention. Addressing these factors holistically may be critical for developing effective, evidence-based adolescent mental health interventions in the digital era.

## Figures and Tables

**Table 1:** Weighted percentages of students with mental health difficulties and their associations with demographic, screen time, and social image factors among U.S. high school students, YRBSS 2019.

				Multivariate Model 1				Multivariate Model 2			
	Unweighted	Weighted	(95% CI)	AOR	(95% CI)	*p*	Adjusted	AOR	(95% CI)	*p*	Adjusted
	N	%					*p*				*p*
Had difficulty concentrating											
Sex											
Female	1934	45.98	(44.12–47.83)	2.00	(1.86–2.17)	<0.0001	0.0004	2.17	(1.97–2.40)	<0.0001	0.0003
Male	1229	29.97	(28.21–31.73)	(reference)				(reference)			
Race/Ethnicity											
Non-Hispanic White	1518	37.10	(35.31–38.90)	(reference)				(reference)			
Non-Hispanic Black	343	31.31	(27.55–35.07)	1.71	(1.42–2.07)	<0.0001	0.0004	0.67	(0.54–0.83)	0.0008	0.0018
Hispanic	892	40.24	(37.61–42.88)	1.13	(0.97–1.31)	0.1271	0.1613	0.98	(0.86–1.12)	0.7456	0.7883
Other	372	41.88	(38.08–45.67)	1.39	(1.16–1.65)	0.0007	0.0017	1.22	(1.01–1.47)	0.0373	0.0627
Grade											
9th	819	35.76	(33.35–38.18)	(reference)				(reference)			
10th	901	39.19	(36.65–41.73)	1.13	(0.96–1.34)	0.1486	0.1751	1.11	(0.96–1.28)	0.1639	0.2374
11th	775	39.91	(37.24–42.59)	1.46	(1.21–1.75)	0.0002	0.0007	1.14	(0.97–1.34)	0.1345	0.2018
12th	672	37.10	(34.32–39.88)	1.56	(1.30–1.88)	<0.0001	0.0004	1.01	(0.82–1.25)	0.9229	0.9454
Television watching											
Less than 2 h	1939	37.03	(35.40–38.66)	(reference)				(reference)			
2–3 h	820	37.44	(34.92–39.96)	1.00	(0.87–1.15)	0.9855	0.9855	0.95	(0.83–1.09)	0.4622	0.5392
4 or more hours	406	45.68	(41.52–49.83)	1.38	(1.17–1.63)	0.0005	0.0014	1.09	(0.91–1.31)	0.3498	0.4452
Digital device use											
Less than 2 h	1036	33.21	(31.16–35.26)	(reference)				(reference)			
2–3 h	910	33.83	(31.59–36.06)	1.09	(0.93–1.27)	0.2673	0.2845	1.11	(0.95–1.29)	0.1919	0.2687
4 or more hours	1216	47.96	(45.54–50.38)	1.91	(1.67–2.18)	<0.0001	0.0004	1.68	(1.47–1.92)	<0.0001	0.0003
Overweight perception											
Yes	253	54.96	(49.37–60.56)					1.30	(1.14–1.49)	0.0004	0.0009
No	2908	36.95	(35.62–38.28)					(reference)			
Sports team participation											
Yes	1590	32.97	(31.32–34.62)					(reference)			
No	1575	44.96	(42.90–47.01)					1.35	(1.22–1.49)	<0.0001	0.0003
Academic grades											
Mostly As and Bs	2122	33.76	(32.30–35.22)					(reference)			
Cs or lower	1011	50.99	(48.29–53.68)					2.19	(1.87–2.56)	<0.0001	0.0003
Slept 5 h or less											
Sex											
Female	1724	26.03	(24.62–27.44)	1.14	(1.01–1.28)	0.0296	0.0465	1.22	(1.07–1.38)	0.0032	0.0064
Male	1522	23.50	(22.10–24.89)	(reference)				(reference)			
Race/Ethnicity											
Non-Hispanic White	1435	22.59	(21.23–23.95)	(reference)				(reference)			
Non-Hispanic Black	595	32.66	(29.61–35.71)	1.71	(1.42–2.07)	<0.0001	0.0004	1.48	(1.23–1.77)	0.0001	0.0003
Hispanic	729	24.57	(22.51–26.62)	1.13	(0.97–1.31)	0.1271	0.1613	0.93	(0.80–1.09)	0.3649	0.4508
Other	431	28.68	(25.69–31.68)	1.39	(1.16–1.65)	0.0007	0.0017	1.37	(1.15–1.64)	0.0011	0.0023
Grade											
9th	763	20.47	(18.72–22.22)	(reference)				(reference)			
10th	830	22.86	(20.96–24.76)	1.13	(0.96–1.34)	0.1486	0.1751	1.10	(0.91–1.32)	0.3368	0.4421
11th	845	27.45	(25.36–29.55)	1.46	(1.21–1.75)	0.0002	0.0007	1.56	(1.27–1.92)	0.0001	0.0003
12th	808	28.76	(26.56–30.95)	1.56	(1.30–1.88)	<0.0001	0.0004	1.66	(1.38–1.98)	<0.0001	0.0003
Television watching											
Less than 2 h	2064	25.68	(24.39–26.97)	(reference)				(reference)			
2–3 h	737	21.17	(19.34–23.00)	0.79	(0.69–0.90)	0.0009	0.002	0.78	(0.68–0.88)	0.0003	0.0007
4 or more hours	401	30.20	(26.90–33.50)	1.20	(1.00-1.43)	0.0526	0.0755	1.03	(0.88–1.20)	0.703	0.777
Digital device use											
Less than 2 h	1277	23.32	(21.76–24.87)	(reference)				(reference)			
2–3 h	829	20.64	(18.99–22.29)	0.88	(0.74–1.05)	0.1637	0.1863	1.00	(0.82–1.21)	0.9585	0.9585
4 or more hours	1143	30.80	(28.84–32.77)	1.45	(1.20–1.76)	0.0003	0.0009	1.43	(1.21–1.68)	<0.0001	0.0003
Overweight perception											
Yes	257	40.61	(35.54–45.67)					1.36	(1.20–1.53)	<0.0001	0.0003
No	2750	24.25	(23.19–25.31)					(reference)			
Sports team participation											
Yes	1144	20.61	(19.25–21.97)					(reference)			
No	1356	31.09	(29.27–32.90)					1.42	(1.23–1.64)	<0.0001	0.0003
Academic grades											
Mostly As and Bs	2124	21.18	(20.09–22.26)					(reference)			
Cs or lower	1120	35.83	(33.61–38.06)					2.19	(1.89–2.53)	<0.0001	0.0003
Felt sad or hopeless											
Sex											
Female	3136	46.65	(45.07–48.23)	2.40	(2.19–2.64)	<0.0001	0.0004	2.43	(2.16–2.74)	<0.0001	0.0003
Male	1728	26.79	(25.34–28.24)	(reference)				(reference)			
Race/Ethnicity											
Non-Hispanic White	2380	36.01	(34.48–37.54)	(reference)				(reference)			
Non-Hispanic Black	627	31.51	(28.60–34.41)	0.82	(0.71–0.96)	0.0139	0.0229	0.78	(0.64–0.95)	0.0164	0.0287
Hispanic	1207	40.00	(37.67–42.32)	1.19	(1.06–1.34)	0.0052	0.0101	1.04	(0.91–1.18)	0.594	0.6743
Other	569	38.42	(35.22–41.62)	1.11	(0.94–1.32)	0.217	0.2387	1.12	(0.94–1.34)	0.2022	0.2739
Grade											
9th	1214	33.24	(31.20–35.27)	(reference)				(reference)			
10th	1358	36.99	(34.86–39.12)	1.17	(0.98–1.41)	0.0843	0.1159	1.18	(0.99–1.40)	0.0607	0.0944
11th	1223	37.89	(35.67–40.11)	1.22	(1.05–1.40)	0.0094	0.0163	1.22	(1.05–1.42)	0.0105	0.02
12th	1080	39.04	(36.66–41.42)	1.28	(1.07–1.53)	0.0083	0.0152	1.22	(0.99–1.49)	0.0568	0.0918
Television watching											
Less than 2 h	2884	35.92	(34.51–37.33)	(reference)				(reference)			
2–3 h	1218	37.07	(34.89–39.26)	1.02	(0.87–1.20)	0.7856	0.8102	0.98	(0.83–1.15)	0.7508	0.7883
4 or more hours	537	42.17	(38.59–45.75)	1.33	(1.10–1.61)	0.0043	0.0089	1.09	(0.87–1.35)	0.4458	0.535
Digital device use											
Less than 2 h	1669	31.95	(30.24–33.66)	(reference)				(reference)			
2–3 h	1347	33.54	(31.62–35.47)	1.18	(1.01–1.39)	0.0367	0.0551	1.24	(1.05–1.47)	0.0141	0.0257
4 or more hours	1759	46.45	(44.34–48.56)	2.00	(1.72–2.33)	<0.0001	0.0004	1.86	(1.55–2.23)	<0.0001	0.0003
Overweight perception											
Yes	354	57.63	(52.55–62.70)					1.32	(1.17–1.49)	<0.0001	0.0003
No	4008	35.77	(34.58–36.95)					(reference)			
Sports team participation											
Yes	1840	32.71	(31.14–34.28)					(reference)			
No	1792	43.34	(41.39–45.30)					1.34	(1.19–1.50)	<0.0001	0.0003
Academic grades											
Mostly As and Bs	3312	34.01	(32.75–35.27)					(reference)			
Cs or lower	1399	46.11	(43.78–48.44)					1.74	(1.53–1.98)	<0.0001	0.0003

Notes. Weighted estimates and 95% CIs account for the YRBSS complex survey design. AOR = adjusted odds ratio. Model 1 adjusted for demographic variables; Model 2 additionally adjusted for screen time and social image factors. Raw and adjusted *p*-values are shown. Adjusted *p*-values were calculated using the Benjamini–Hochberg false discovery rate (FDR) correction across all 42 *p*-values in the table.

**Table 2:** Weighted percentage of students who reported suicidal ideation and behavior and their associations with demographic, screen time, and social image factors among U.S. high school students, YRBSS 2019.

				Multivariate Model 1				Multivariate Model 2			
	Unweighted	Weighted	(95% CI)	AOR	(95% CI)	*p*	Adjusted	AOR	(95% CI)	*p*	Adjusted
	N	%					*p*				*p*
Considered Suicide											
Sex											
Female	1691	24.09	(22.74–25.44)	2.09	(1.85–2.36)	<0.0001	0.0006	2.17	(1.85–2.54)	<0.0001	0.0004
Male	897	13.31	(12.22–14.41)	(reference)				(reference)			
Race/Ethnicity											
Non-Hispanic White	1273	19.15	(17.87–20.42)	(reference)				(reference)			
Non-Hispanic Black	354	16.89	(14.57–19.21)	0.88	(0.75–1.03)	0.1026	0.224	0.80	(0.65–0.98)	0.0347	0.0662
Hispanic	563	17.23	(15.51–18.95)	0.87	(0.74–1.03)	0.1086	0.224	0.71	(0.58–0.88)	0.0023	0.0074
Other	362	23.05	(20.34–25.77)	1.28	(1.05–1.56)	0.0168	0.0554	1.27	(1.02–1.57)	0.0334	0.0662
Grade											
9th	702	17.65	(16.07–19.23)	(reference)				(reference)			
10th	723	18.49	(16.81–20.16)	1.06	(0.85–1.32)	0.599	0.6589	1.10	(0.88–1.37)	0.3785	0.4726
11th	639	19.32	(17.51–21.13)	1.10	(0.92–1.31)	0.2855	0.4959	1.10	(0.91–1.34)	0.3185	0.418
12th	538	19.63	(17.66–21.60)	1.10	(0.90–1.34)	0.3403	0.5348	1.11	(0.90–1.37)	0.3044	0.4124
Television watching											
Less than 2 h	1555	18.89	(17.74–20.05)	(reference)				(reference)			
2–3 h	613	16.58	(14.99–18.17)	1.02	(0.87–1.20)	0.79	0.79	0.85	(0.71–1.00)	0.0551	0.0926
4 or more hours	313	23.72	(20.69–26.74)	1.33	(1.10–1.61)	<0.0001	0.0006	1.09	(0.87–1.35)	0.4474	0.522
Digital device use											
Less than 2 h	876	15.83	(14.51–17.15)	(reference)				(reference)			
2–3 h	705	16.13	(14.70–17.56)	1.18	(1.01–1.39)	0.04	0.1015	1.07	(0.90–1.27)	0.4234	0.5081
4 or more hours	970	25.24	(23.39–27.08)	2.00	(1.72–2.33)	<0.0001	0.0006	1.69	(1.37–2.08)	<0.0001	0.0004
Overweight perception											
Yes	259	37.58	(32.68–42.47)					1.67	(1.43–1.95)	<0.0001	0.0004
No	2089	17.86	(16.92–18.80)					(reference)			
Sports team participation											
Yes	960	15.66	(14.51–16.81)					(reference)			
No	956	23.25	(21.55–24.94)					1.37	(1.15–1.63)	0.001	0.0035
Academic grades											
Mostly As and Bs	1748	17.04	(16.06–18.02)					(reference)			
Cs or lower	780	24.56	(22.54–26.58)					1.68	(1.49–1.89)	<0.0001	0.0004
Planned suicide											
Sex											
Female	1366	19.95	(18.68–21.21)	1.94	(1.74–2.16)	<0.0001	0.0006	2.05	(1.78–2.36)	<0.0001	0.0004
Male	748	11.33	(10.30–12.36)	(reference)				(reference)			
Race/Ethnicity											
Non-Hispanic White	996	15.69	(14.50–16.89)	(reference)				(reference)			
Non-Hispanic Black	312	15.02	(12.90–17.15)	0.97	(0.78–1.20)	0.7856	0.79	0.84	(0.64–1.08)	0.1676	0.2514
Hispanic	482	14.74	(13.15–16.34)	0.92	(0.77–1.11)	0.3799	0.554	0.75	(0.61–0.92)	0.0085	0.0238
Other	292	19.17	(16.56–21.78)	1.29	(1.05–1.59)	0.0186	0.0558	1.31	(1.06–1.63)	0.0164	0.0383
Grade											
9th	573	14.77	(13.31–16.24)	(reference)				(reference)			
10th	607	15.39	(13.84–16.94)	1.04	(0.85–1.28)	0.6884	0.7328	1.10	(0.89–1.36)	0.3826	0.4726
11th	511	16.4	(14.64–18.16)	1.11	(0.90–1.37)	0.3054	0.5039	1.11	(0.88–1.40)	0.0085	0.0238
12th	433	16.2	(14.39–18.01)	1.07	(0.87–1.33)	0.5047	0.5948	1.11	(0.90–1.38)	0.0164	0.0383
Television watching											
Less than 2 h	1273	15.61	(14.55–16.67)	(reference)				(reference)			
2–3 h	510	15.16	(13.55–16.78)	0.94	(0.78–1.13)	0.4788	0.5852	0.95	(0.78–1.17)	0.6204	0.6536
4 or more hours	260	19.36	(16.60–22.13)	1.26	(0.99–1.60)	0.0564	0.1329	1.01	(0.76–1.36)	0.9204	0.9204
Digital device use											
Less than 2 h	711	13.06	(11.86–14.27)	(reference)				(reference)			
2–3 h	571	13.85	(12.45–15.24)	1.14	(0.96–1.35)	0.1275	0.2338	1.21	(1.00–1.47)	0.0531	0.0926
4 or more hours	813	21.30	(19.56–23.04)	1.87	(1.57–2.23)	<0.0001	0.0006	1.77	(1.44–2.18)	<0.0001	0.0004
Overweight perception											
Yes	206	31.7	(26.98–34.42)					1.61	(1.38–1.88)	<0.0001	0.0004
No	1717	14.96	(14.09–15.84)					(reference)			
Sports team participation											
Yes	822	14.24	(13.09–15.40)					(reference)			
No	773	18.71	(17.16–20.25)					1.16	(1.01–1.33)	0.0416	0.076
Academic grades											
Mostly As and Bs	1393	14.05	(13.14–14.97)					(reference)			
Cs or lower	674	21.5	(19.58–23.43)					1.81	(1.52–2.14)	<0.0001	0.0004
Attempted suicide											
Sex											
Female	664	11.04	(9.98–12.10)	1.76	(1.39–2.22)	<0.0001	0.0006	1.90	(1.43–2.51)	<0.0001	0.0004
Male	374	6.64	(5.75–7.53)	(reference)				(reference)			
Race/Ethnicity											
Non-Hispanic White	446	7.90	(6.96–8.84)	(reference)				(reference)			
Non-Hispanic Black	183	11.85	(9.54–14.16)	1.57	(1.16–2.12)	0.0047	0.0194	1.44	(1.06–1.98)	0.0229	0.0506
Hispanic	254	8.91	(7.53–10.29)	1.10	(0.84–1.44)	0.465	0.5852	0.85	(0.62–1.16)	0.2912	0.4102
Other	135	10.98	(8.66–13.29)	1.44	(1.05–1.96)	0.0242	0.0666	1.53	(1.10–2.13)	0.0125	0.0328
Grade											
9th	316	9.41	(8.13–10.69)	(reference)				(reference)			
10th	307	8.79	(7.48–10.10)	0.94	(0.77–1.15)	0.5405	0.6151	0.94	(0.74–1.20)	0.6225	0.6536
11th	237	8.62	(7.18–10.05)	0.91	(0.71–1.16)	0.424	0.583	0.86	(0.65–1.14)	0.293	0.4102
12th	189	8.49	(6.94–10.03)	0.88	(0.67–1.18)	0.3861	0.554	0.93	(0.70–1.25)	0.6162	0.6536
Television watching											
Less than 2 h	626	8.57	(7.68–9.46)	(reference)				(reference)			
2–3 h	229	7.65	(6.39–8.91)	0.84	(0.67–1.05)	0.1214	0.2338	0.82	(0.63–1.07)	0.1378	0.2144
4 or more hours	139	13.21	(10.59–15.83)	1.46	(1.12–1.90)	0.0063	0.0231	1.09	(0.80–1.47)	0.5875	0.6536
Digital device use											
Less than 2 h	379	8.09	(6.99–9.18)	(reference)				(reference)			
2–3 h	280	7.65	(6.07–8.18)	0.93	(0.75–1.15)	0.4787	0.5852	0.95	(0.74–1.23)	0.7093	0.7266
4 or more hours	365	13.21	(9.81–12.71)	1.54	(1.21–1.97)	0.0011	0.0052	1.31	(0.99–1.74)	0.0624	0.1008
Overweight perception											
Yes	103	21.19	(16.46–25.92)					1.60	(1.31–1.95)	<0.0001	0.0004
No	820	8.15	(7.42–8.87)					(reference)			
Sports team participation											
Yes	470	7.58	(6.72–8.44)					(reference)			
No	426	10.77	(9.44–12.10)					1.24	(1.03–1.50)	0.0266	0.0559
Academic grades											
Mostly As and Bs	635	6.77	(6.07–7.47)					(reference)			
Cs or lower	378	15.55	(13.65–17.46)					2.54	(1.99–3.23)	<0.0001	0.0004

Notes. Weighted estimates and 95% CIs account for the YRBS complex survey design. AOR = adjusted odds ratio. Model 1 adjusted for demographic variables; Model 2 additionally adjusted for screen time and social image factors. Raw and adjusted *p*-values are shown. Adjusted *p*-values were calculated using the Benjamini–Hochberg false discovery rate (FDR) correction across all 42 *p*-values in the table.

**Table 3:** Weighted percentages of students who spent 4 or more hours of screen time per day and their associations with demographic and social image factors among U.S. high school students, YRBSS 2019.

	Unweighted	Weighted	(95% CI)	AOR	(95% CI)	*p*	Adjusted
	N	%					*p*
Television watching ≥ 4 h							
Sex							
Female	702	10.7	(9.68–11.71)	1.26	(1.07–1.48)	0.0076	0.019
Male	631	9.67	(8.70–10.64)	(reference)			
Race/Ethnicity							
Non-Hispanic White	447	7.30	(6.45–8.15)	(reference)			
Non-Hispanic Black	380	19.9	(17.37–22.43)	3.01	(2.45–3.71)	<0.0001	0.0004
Hispanic	320	11.33	(9.76–12.90)	1.30	(1.18–1.77)	0.0007	0.0023
Other	156	9.76	(7.90–11.62)	1.54	(1.07–2.23)	0.023	0.046
Grade							
9th	388	11.23	(9.82–12.64)	(reference)			
10th	387	10.77	(9.34–12.20)	0.91	(0.76–1.09)	0.2989	0.427
11th	293	9.58	(8.21–10.96)	0.79	(0.61–1.03)	0.0771	0.1285
12th	259	8.83	(7.46–10.21)	0.77	(0.61–0.97)	0.0255	0.0464
Overweight perception							
Yes	97	12.69	(9.54–15.84)	1.14	(1.02–1.27)	0.0194	0.0431
No	1142	9.98	(9.24–10.72)	(reference)			
Sports team participation							
Yes	512	8.53	(7.63–9.43)	(reference)			
No	515	12.20	(10.87–13.52)	1.37	(1.15–1.63)	0.0008	0.0023
Academic grades							
Mostly As and Bs	835	8.44	(7.70–9.19)	(reference)			
Cs or lower	459	15.06	(13.38–16.75)	1.78	(1.41–2.23)	<0.0001	0.0004
Digital device use ≥ 4 h							
Sex							
Female	1969	30.9	(29.40–32.39)	0.98	(0.88–1.10)	0.7533	0.8862
Male	1905	30.52	(28.99–32.06)	(reference)			
Race/Ethnicity							
Non-Hispanic White	1812	29.17	(27.68–30.65)	(reference)			
Non-Hispanic Black	624	34.40	(31.33–37.47)	1.21	(0.93–1.57)	0.1528	0.2351
Hispanic	925	31.90	(29.65–34.15)	1.06	(0.92–1.22)	0.4261	0.5681
Other	443	30.72	(27.67–33.78)	1.08	(0.87–1.34)	0.4836	0.6045
Grade							
9th	1049	30.36	(28.33–32.39)	(reference)			
10th	1104	31.2	(29.10–33.31)	0.99	(0.83–1.17)	0.8901	0.9369
11th	903	30.66	(28.49–32.83)	1.02	(0.85–1.23)	0.8184	0.9093
12th	821	30.48	(28.20–32.75)	1.00	(0.80–1.26)	0.9779	0.9779
Overweight perception							
Yes	273	43.50	(38.37–48.63)	1.44	(1.29–1.60)	<0.0001	0.0004
No	3252	29.95	(28.80–31.09)	(reference)			
Sports team participation							
Yes	1429	25.68	(24.21–27.15)	(reference)			
No	1542	38.68	(36.74–40.61)	1.68	(1.44–1.97)	<0.0001	0.0004
Academic grades							
Mostly As and Bs	2646	27.85	(26.64–29.05)	(reference)			
Cs or lower	1178	39.58	(37.28–41.88)	1.48	(1.30–1.68)	<0.0001	0.0004

Notes. Weighted estimates and 95% CIs account for the YRBSS complex survey design. AOR = adjusted odds ratio. Models were adjusted for demographic variables and social image factors. Raw and adjusted *p*-values are shown. Adjusted *p*-values were calculated using the Benjamini–Hochberg false discovery rate (FDR) correction across all 20 *p*-values in the table.

## Data Availability

The data are publicly available and can be downloaded from the Centers for Disease Control and Prevention (CDC): https://www.cdc.gov/yrbs/data/index.html, accessed on 24 August 2024.

## Supplementary Materials

The following supporting information can be downloaded at: https://www.mdpi.com/article/10.3390/healthcare13222833/s1, Table S1: STROBE Checklist for the 2019 Youth Risk Behavior Surveillance System (YRBSS) Cross-Sectional Study.

## Abbreviations

The following abbreviations are used in this manuscript:

YRBSS	Youth Risk Behavior Surveillance System
CDC	Centers for Disease Control and Prevention
AOR	Adjusted Odds Ratio
CI	Confidence Interval
AI	Artificial Intelligence
