# Supplementary material for: Excessive Screen Time Among U.S. High School Students: Mental Health, Suicidal Ideation and Social Image Factors
Source: Healthcare (Basel). 2025 Nov 8;13(22):2833. doi: 10.3390/healthcare13222833 (PMC12652926; doi:10.3390/healthcare13222833)
Supplement: Supplementary file 1 [file healthcare-13-02833-s001.zip › healthcare-3877594-supplementary.pdf]

Table S1 STROBE Checklist for the 2019 Youth Risk Behavior Surveillance System (YRBSS) Cross-Sectional Study

Item No.	Recommendation	Section of Manuscript	Page No.
1(a)	Indicate the study's design with a commonly used term in the title or abstract.	Title, Abstract	1
1(b)	Provide an informative and balanced summary of what was done and what was found.	Abstract	1
2	Explain the scientific background and rationale for the investigation.	Introduction, Paragraphs 1–3	1-2
3	State specific objectives, including any prespecified hypotheses.	Introduction	2-4
4	Present key elements of study design early in the paper.	Introduction, the last three paragraph	3-4
5	Describe the setting, locations, and relevant dates.	Methods, "Data Source and Population"	4

6(a)	Give eligibility criteria, and sources/methods of participant selection.	Methods, “Population”	4
7	Clearly define all outcomes, exposures, predictors, confounders, and effect modifiers.	Methods, “Outcome Measures” “Independent Variables” “Demographic and Moderating Variables”	4-5
8	For each variable, give data sources and details of assessment (measurement).	Methods, “Data Source and Population”	4
9	Describe any efforts to address potential sources of bias.	Discussion, “Limitations”	17
10	Explain how the study size was arrived at.	Methods, “Data Source and Population”	4
11	Explain how quantitative variables were handled in the analyses.	Methods, “Statistical Analysis”	5
12(a)	Describe all statistical methods, including those	Methods, “Statistical Analysis”	5

	used to control for confounding.		
12(b)	Describe any methods used to examine subgroups and interactions.	Methods, “Interaction Terms”	7
12(c)	Explain how missing data were addressed.	Methods, “Data Analysis”	7
12(d)	Describe analytical methods accounting for sampling strategy.	Methods, “Data Analysis”	7
12(e)	Describe any sensitivity analyses.	Results	6
13(a)	Report numbers of individuals at each stage of study.	Results, “Participant Characteristics”	6
13(b)	Give reasons for non-participation at each stage.	N/A (secondary data)	—
14(a)	Give characteristics of study participants and information on exposures and confounders.	Results	6
14(b)	Indicate number of participants	Results, “Participants	

	with missing data for each variable.	Characteristics" (summary only)	
15	Report numbers of outcome events or summary measures.	Results, Tables 1–3	7-8, 11-13
16(a)	Give unadjusted and adjusted estimates with precision (e.g., 95% CI).	Results, Tables 1–3	7-8, 11-13
16(b)	Report category boundaries when continuous variables were categorized.	Methods	6
16(c)	If relevant, consider translating estimates of relative risk into absolute risk.	Not applicable (cross-sectional)	—
18	Summarize key results with reference to study objectives.	Discussion, first paragraph	14-15
19	Discuss limitations of the study, considering potential bias or imprecision.	Discussion, "Limitations"	17
20	Give cautious overall interpretation	Discussion "Limitations"	17

	considering multiplicity of analyses and prior evidence.		
21	Discuss generalizability (external validity) of the results.	Discussion (Limitation)	17
22	Give the source of funding and role of funders.	Not applicable	

This STROBE checklist was completed based on the finalized manuscript and indicates where each item is addressed within the text.
